# Biofilm Matrix and Its Regulation in *Pseudomonas aeruginosa*

**DOI:** 10.3390/ijms141020983

**Published:** 2013-10-18

**Authors:** Qing Wei, Luyan Z. Ma

**Affiliations:** State Key Laboratory of Microbial Resources, Institute of Microbiology, Chinese Academy of Sciences, No.3, 1st Beichen West Road, Chaoyang District, Beijing 100101, China; E-Mail: vubwqing@hotmail.com

**Keywords:** *Pseudomonas aeruginosa*, biofilm matrix, exopolysaccharides, gene regulation, anti-biofilm

## Abstract

Biofilms are communities of microorganisms embedded in extracellular polymeric substances (EPS) matrix. Bacteria in biofilms demonstrate distinct features from their free-living planktonic counterparts, such as different physiology and high resistance to immune system and antibiotics that render biofilm a source of chronic and persistent infections. A deeper understanding of biofilms will ultimately provide insights into the development of alternative treatment for biofilm infections. The opportunistic pathogen *Pseudomonas aeruginosa*, a model bacterium for biofilm research, is notorious for its ability to cause chronic infections by its high level of drug resistance involving the formation of biofilms. In this review, we summarize recent advances in biofilm formation, focusing on the biofilm matrix and its regulation in *P. aeruginosa*, aiming to provide resources for the understanding and control of bacterial biofilms.

## 1. Introduction

Biofilms are microbial communities encased in extracellular polymeric substances (EPS) [1]. Biofilm formation represents a protective mode of growth that allows microorganisms to survive in hostile environments and disperse seeding cells to colonize new niches under desirable conditions. Biofilms can form on a variety of surfaces and are prevalent in natural, industrial, and hospital niches. These sessile microbial communities are physiologically distinct from free-living planktonic counterparts [2,3]. Clinically, biofilms are responsible for many persistent and chronic infections due to their inherent resistance to antimicrobial agents and the selection for phenotypic variants. A better understanding of the genetic and molecular mechanisms of biofilm formation may provide strategies for the control of chronic infections and problems related to biofilm formation.

The EPS of biofilm is a mixture of polysaccharides, extracellular DNA (eDNA), and proteins, which function as matrix, or glue, holding microbial cells together. The biofilm matrix contributes to the overall architecture and the resistance phenotype of biofilms [4,5]. Uncovering roles played by EPS matrices in biofilm formation will be beneficial for the design of targeted molecules to control biofilm formation. In this review, advances in biofilm formation and regulation are presented with a focus on the biofilm matrix in *P. aeruginosa*, a model organism for biofilm research.

## 2. Matrices of *P. aeruginosa* Biofilms

Colonization of the lungs of cystic fibrosis (CF) patients by *P. aeruginosa* is the major cause of morbidity and mortality in CF populations. These infections generally persist despite the use of long term aggressive antimicrobial therapy and have been associated with the formation of antibiotic-resistant biofilms, whereby bacterial communities form microcolonies embedded in a hydrated EPS matrix [6,7]. The relative importance of the EPS matrix is dependent on the genetic background of strains, nutritional conditions and developmental phases of biofilms [8]. It is generally acknowledged that the EPS of biofilms functions as both a structural scaffold and/or a protective barrier to harsh environments [9,10]. At least three polysaccharides (Psl, Pel and alginate) have been identified in *P. aeruginosa* that play important roles in structure maintenance and antibiotic resistance of biofilm [9,11–15]. The respective nature and functions in biofilm formation and development of biofilm matrix components including exopolysaccharides (Psl, Pel, and alginate), eDNA, proteins, and proteinaceous surface appendages such as fimbriae, type IV pili (T4P), and flagellum will be discussed below.

### 2.1. Psl Polysaccharide

Polysaccharide synthesis locus of *P. aeruginosa* PAO1 was identified in 2004 by three different groups [11,13,16]. The Psl cluster consists of 15 co-transcribed genes (*pslA* to *pslO*, PA2231-2245) encoding proteins to synthesize Psl, which is important to initiate and maintain biofilm structure by providing cell-cell and cell-surface interactions [15,17–19]. It was further demonstrated that only 11 of the 15 *psl* genes are required for the synthesis of Psl-dependent biofilm [20]. A deletion from *pslA* to *pslD* in *P. aeruginosa* strain PA14 leads to the incapability of producing Psl polysaccharide [21]. It was previously shown that Psl is a galactose-rich and mannose-rich exopolysaccharide with relatively lower amounts of glucose and xylose [17]. Recently, Psl was found to contain a repeating pentasaccharide consisting of d-mannose, d-glucose, and l-rhamnose [20]. Additionally, functions of individual *psl* genes such as *pslA*, *pslB* and *pslD* have been studied at genetic and biochemical levels, revealing that those genes are crucial for the biofilm formation and Psl synthesis [22–24].

**Figure 1:**
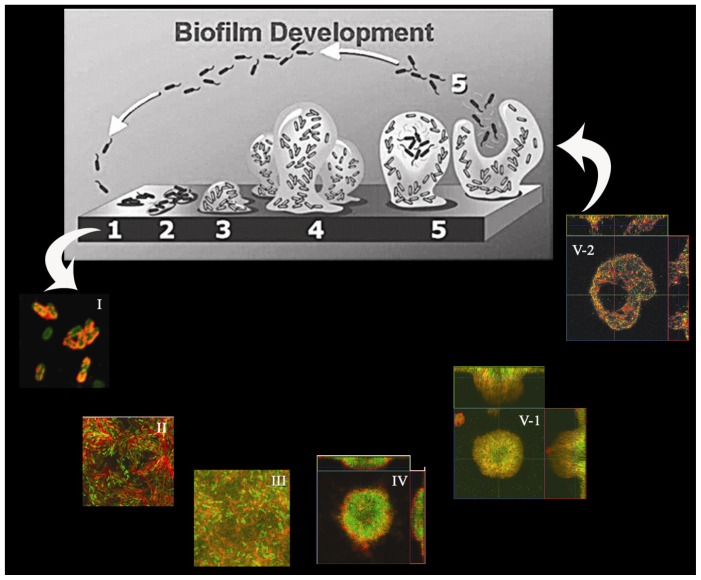
Scheme of biofilm development in *P. aeruginosa*. Selected images showed how the matrix of Psl polysaccharide (**red** fluorescence) enmeshes bacterial cells (**green** fluorescence) within bacterial communities during biofilm development (**I**: initial attachment; **II**: irreversible attachment; **III**: microcolony formation; **IV**: biofilm maturation; **V**: biofilm dispersion). The figure was used with the permission of the authors [15,27] and modified herein.

The roles of Psl in biofilm formation have been thoroughly investigated due to its major contribution to the biofilm formation in *P. aeruginosa* [19]. Firstly, overproduction of the Psl polysaccharide led to enhanced cell-surface and intercellular adhesion of *P. aeruginosa*, suggesting its importance in adhesion, which is critical for initiation and maintenance of the biofilm structure [18,20]. Afterwards, using fluorescently labeled lectins, Ma and colleagues could directly visualize Psl exopolysaccharide formation at different stages of biofilm development (Figure 1). They found that Psl polysaccharide anchors on bacterial cell surface in a helical shape, which promotes strong bacterial cell-cell interactions. This results in the assembly of a biofilm and its matrix at the early stage of biofilm development. Later on, Psl polysaccharide accumulated on the periphery of three dimension-structured macrocolonies during biofilm maturation. This localization pattern provided the structure support and allowed for later biofilm dispersion [15]. In addition, Psl staining demonstrated that Psl can form a fiber-like matrix that enmeshes bacteria within biofilms. Recently, it was found that Psl fiber matrix was formed via a T4P-dependent migration strategy, a way similar to spider web formation [25]. Independently, Zhao and colleagues also showed that *P. aeruginosa* can deposit Psl trails during migration on a surface, which influences the surface motility of subsequent cells, leading to the biofilm initiation [26]. These recent discoveries further expand our understanding on the biology of Psl polysaccharide.

Strikingly, the function of Psl was further characterized to have a signaling role in stimulating two diguanylate cyclase, SiaD and SadC, to produce more of the intracellular second messenger molecule c-di-GMP, thus ultimately increasing the production of Psl itself and forming a unique positive feedback regulatory circuit [28].

In addition, Psl was found to have roles in pathogenesis and protection against the immune system. It was shown that by increasing contact between bacterial cells and epithelial cells, Psl polysaccharide indirectly stimulates NF-κB activity and facilitates flagellin-mediated proinflammatory signaling [29]. Furthermore, Mishra *et al.* found that *P. aeruginosa* Psl polysaccharide could reduce neutrophil phagocytosis and the oxidative response via limiting complement-mediated opsonization [30]. This study clearly presented Psl as a survival advantage *in vivo* and provided evidence for the clearance of persistent infections using Psl as a target.

Reports showed that Psl also had roles in antibiotics resistance. It was recently revealed that Psl can promote resistance to the biofilm inhibitor Polysorbate 80 [31]. Yang and colleagues reported that the formation of tight microcolony structure, mainly by Psl, in *P. aeruginosa* contributed to the resistance to antibiotic treatment [19]. By using fluorescent labeling of antibiotics, the extracellular matrix was also found to protect *P. aeruginosa* biofilms from killing by limiting the penetration of tobramycin; this sequestration happened at the periphery of biofilms [32]. However, no clear evidence was identified in which biofilm matrix is critical for this protection and interaction. Furthermore, another group showed that Psl provided a generic first line of defense against both cationic and anionic antibiotics during the initial stages of biofilm development and the Psl-mediated protection was extendable to *Escherichia coli* and *Staphylococcus aureus* in co-culture biofilms [33].

In summary, Psl not only functions as a scaffold for biofilm development, but also serves as a signaling molecule or track for the subsequent events leading to the formation of biofilms. This positive feedback circuit presents an evolutionary survival advantage for *P. aeruginosa* to colonize different niches. Besides, Psl also functions as a barrier for immune and antibiotic attacks.

### 2.2. Pel Polysaccharide

Pel polysaccharide, which is synthesized by the products of the *pel* gene cluster (*pelA-F*, PA3058-PA3064), is a glucose-rich and cellulase-sensitive extracellular matrix [12]. This gene cluster is conserved in other Gram-negative bacteria [34]. A *pel* mutant appeared to be deficient in the formation of pellicles at the air-liquid interface in standing cultures. Pel is also required for the formation of solid surface-associated biofilms [11]. Interestingly, in a non-piliated *P. aeruginosa* PAK strain, the *pel* mutation was discovered to have severe defects in the initial attachment process on solid surfaces, suggesting that Pel polysaccahride can compensate as an attachment factor in the absence of other adhesins such as type IV pili [34]. However, the precise role of Pel polysaccharide in attachment in other *P. aeruginosa* strains needs further investigation. In addition, Pel polysaccharide can serve as a primary structure scaffold for the community of cells by maintaining the cell-to-cell interactions in PA14 biofilms and play a protective role by enhancing resistance to aminoglycoside antibiotics in biofilms [14]. The role of Pel in biofilm formation was further investigated and it was revealed that Pel could function together with other types of EPS throughout biofilm development in *P. aeruginosa* PAO1, although in a less important role as compared to Psl. [19].

One remaining question about Pel is its biochemical composition. Although the original study identified Pel as a glucose-rich matrix material [12], its defined structure and feature have not been completely understood. Recently, the main glucose-containing carbohydrate of the extracellular matrix of *P. aeruginosa* PA14 was characterized to be glycerophosphorylated cyclic β-(1,3)-glucans, which was synthesized by *ndvB* locus but not the *pel* operon [35]. A systematic analysis of the extracellular carbohydrates produced by *P. aeruginosa* PA14 elucidated the structure of a LPS *O*-antigen polysaccharide, which suggested that the *pel* locus might be involved in the production of the LPS [36]. Recently, biochemical studies identified PelF as a cytosolic glycosyltransferase that utilizes UDP-glucose as substrate for Pel synthesis [37]. In addition, Colvin and his coworkers examined the deacetylase activity of PelA and found that this function is important for the production of the Pel polysaccharide [38]. Both studies contributed to the understanding of the synthesis of Pel in *P. aeruginosa.*

### 2.3. Alginate

Alginate is the exopolysaccharide that is often and mainly produced by *P. aeruginosa* clinical isolates from the lungs of CF patients [39]. The typical mucoid phenotype is due to the overproduction of this polysaccharide, which protects *P. aeruginosa* from harsh environments in CF lungs by providing an extracellular matrix in biofilms. However, it is not absolutely required during the formation of nonmucoid biofilms *in vitro* [40]. Alginate plays important roles in structural stability and protection of biofilms. It is necessary for water and nutrient retention in biofilms [41]. Interestingly, it was recently found that mucoid *P. aeruginosa* strains also relied on Psl to form biofilms [42,43]. Alginate has been identified to have functions in persistence and immune evasion [44]. Overproduction of alginate could provide resistance to antibiotics as well as opsonophagocytosis [45,46]. Alginate also has the ability to scavenge free radicals released from neutrophils and activate macrophages *in vitro* that are commonly used to kill pathogens [47]. Bragonzi and colleagues reported that overproduction of alginate did not provide increased persistence in a murine lung infection model, perhaps likely due to the reversion of mucoid phenotype to non-mucoid phenotype during infections [48].

### 2.4. Extracellular DNA

Extracellular DNA (eDNA) constitutes another important component of the *P. aeruginosa* biofilm matrix [16,49,50]. The eDNA appears to be generated from random chromosomal DNA that serves as a cell-to-cell interconnecting component in the biofilm. Biofilm eDNA staining suggests that the eDNA is located primarily in high concentrations within the stalks of mushroom-shaped microcolonies [49]. In addition, bacterial cells also undergo autolysis in biofilm microcolonies, but it is unclear whether autolysis contributes to eDNA or biofilm development [51]. Several hypotheses have been proposed such as direct secretion, lysis of sub-population by prophage and release of small membrane vesicles [52].

Similar to other types of biofilm matrices, eDNA also has multifaceted roles in biofilm formation, such as contribution to cation gradients, genomic DNA release and antibiotic resistance [53]. eDNA also acts as a nutrient source for bacteria during starvation [54]. Interestingly, it was recently shown that *P. aeruginosa* produces an extracellular deoxyribonuclease (PA3909) that is required for utilization of eDNA as a nutrient source, further amplifying the role of eDNA during *P. aeruginosa* biofilm formation [55]. In addition, eDNA was shown to form bundles with F-actin liberated from necrotic neutrophils and the presence of those bundles could stimulate the initial development of *P. aeruginosa* biofilms [56]. eDNA can also activate neutrophils through a CpG- and TLR9-independent mechanism and serve as a major proinflammatory component of *P. aeruginosa* biofilms [57]. eDNA was further found to facilitate the twitching motility-mediated biofilm expansion by maintaining coherent cell alignments to coordinate the movement of cells in the leading edge vanguard rafts [58].

### 2.5. Proteins and Proteinaceous Bacterial Surface Appendages

Aside from exopolysaccharides and eDNA, extracellular proteins and several proteinaceous components are also considered to be matrix components, including type IV pili, flagella, and fimbriae. These components were found to mainly play auxiliary functions as adhesion factors and structural support in the biofilm formation of *P. aeruginosa* [59].

Flagella mediate swimming and swarming motility of *P. aeruginosa*. It can also act as an adhesin and play critical roles in the initial cell-to-surface interactions [60]. T4P is a linear actuator critical for twitching motility that involves an extension-grip-retraction mechanism [61]. T4P plays important roles in microcolony formation of *P. aeruginosa* biofilms by forming typical mushroom caps [60]. As mentioned above, T4P-driven bacterial motilities can help to form Psl fiber matrix [25]. Another group, however, drew the conclusion that flagella and pili are not required for the initial attachment or biofilm formation [62], indicating that flagella- and pili-mediated biofilm formation could be conditional and nutritional. Recently, another newly identified adhesin named CdrA was demonstrated to be a key protein functioning as a structural component of *P. aeruginosa* EPS matrix [63]. This adhesin could directly interact with Psl polysaccharide to mediate bacterial auto-aggregation and increase biofilm stability.

The *P. aeruginosa* Cup fimbriae constitute one class of appendages that facilitate the biofilm formation and assemble through chaperone/usher pathway [64]. In *P. aeruginosa* PAO1, at least four Cup systems (CupA, B, C, and E) have been identified and vary in organizations and functions [65]. It was demonstrated that Cup fimbriae are critical for the initial stage of biofilm development, particularly in cell-to-cell interaction and microcolony formation [66,67].

Numerous investigations on biofilm formation, especially the biofilm matrices of *P. aeruignosa* have provided insights into the importance and nature of each matrix component and laid foundations for the efficient treatment of biofilm-related *P. aeruginosa* infections. The roles of matrix components in *P. aeruginosa* biofilms are summarized in Table 1.

## 3. Regulation of Biofilm Matrix in *P. aeruginosa*

Gene regulation is important for our understanding of biofilm formation. Generally, organisms form a biofilm in response to several factors including nutritional cues, secondary messengers, host-derived signals or, in some cases, to sub-inhibitory concentrations of antibiotics [1,68]. When a cell switches to the biofilm mode of growth, it undergoes a phenotypic shift in behavior whereby a large array of genes is differentially regulated [69].

Biofilm formation is a multicellular process involving environmental signals and a concerted regulation combining both environmental signals and regulatory networks. Due to the major roles of EPS matrix in biofilm formation, its regulation is discussed.

### 3.1. c-di-GMP

**Figure 2:**
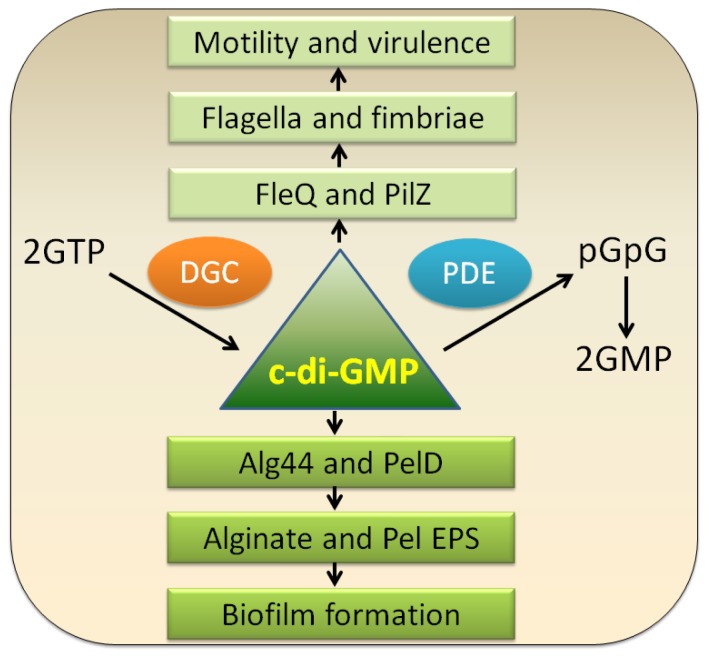
Schematic presentation of physiological functions of c-di-GMP. In bacterial cells, c-di-GMP is generated by diguanylate cyclases (DGC) and broken down by specific phosphodiesterases (PDE). As a second messenger, low levels of c-di-GMP can promote motility by upregulating flagellar expression, assembly or interfering with flagellar motor function and are required for the expression of acute virulence genes. High levels of c-di-GMP however favor sessility and stimulate the synthesis of various matrix exopolysaccharides, such as Pel (mediated by PelD) and alginate (mediated by Alg44) [71,74,75].

Bis-(3′-5′)-cyclic dimeric guanosine monophosphate (c-di-GMP), a ubiquitous intracellular second messenger widely distributed in bacteria, was discovered in 1987 as an allosteric activator of the cellulose synthase complex in *Gluconacetobacter xylinus* [70]. In general, c-di-GMP stimulates the biosynthesis of adhesins and exopolysaccharide mediated biofilm formation and inhibits bacterial motilities, which controls the switch between the motile planktonic and sessile biofilm-associated lifestyle of bacteria (Figure 2). Moreover, c-di-GMP controls the virulence of animal and plant pathogens, progression through the cell cycle, antibiotic production and other cellular functions [71–73].

C-di-GMP is synthesized from two molecules of GTP by diguanylate cyclases (DGC) containing GGDEF domains and is broken down into 5′-phosphoguanylyl-(3′-5′)-guanosine (pGpG) by specific phosphodiesterases (PDE) containing EAL or HD-GYP domains; pGpG is subsequently split into two GMP molecules (Figure 2). These conserved domains are essential for their corresponding enzymatic activities [71,73]. Whole genome sequencing analysis has revealed that *E. coli* has 19 GGDEF and 17 EAL domain proteins while *Vibrio vulnificus* encodes up to 100 of those proteins [73]. In *P. aeruginosa*, there are 41 of such proteins, including 3 HD-GYP, 17 GGDEF, and 5 EAL domain proteins, as well as 16 proteins with both GGDEF and EAL domains [76,77]. Most proteins that contain these domains show a multi-modular arrangement. In addition to GGDEF, EAL, or HD-GYP domains, there are a variety of sensory domains such as signal receiver or transmission domains and response regulator domains that are likely to receive signals from the environment [73]. These signals are proposed to be translated as an alteration of the enzymatic activity that would result in local or global fluctuations in c-di-GMP levels, which in turn would lead to behavioral adjustments [1].

The mechanism of c-di-GMP signal transduction generally involves the first recognition of c-di-GMP signal and the subsequent phenotypic regulation. To exert its function, c-di-GMP binds to effectors (c-di-GMP receptors), and by allostery, alters their structure and output function [71]. Those c-di-GMP effectors are highly diverse, among which the PilZ family of proteins are the best-studied. In *P. aeruginosa*, at least four c-di-GMP effectors are present including FleQ, PelD, Alg44, and PilZ. FleQ is a c-di-GMP-binding transcription factor, which generally functions as an activator in flagella biosynthesis. Yet it can also act as a repressor for the transcription of the *pel* gene cluster in the absence of c-di-GMP and an activator upon c-di-GMP binding [78]. PelD is part of *pel* operon of *P. aeruginosa* activated by direct binding c-di-GMP through a site that resembles the I site motif in GGDEF domain proteins [79]. Alg44 is another PilZ family protein involved in alginate synthesis [80]. PilZ is a type IV fimbrial biogenesis protein involved in twitching [81]. However, it is still a mystery if there is any receptor protein of c-di-GMP accounting for Psl polysaccharide production.

Intriguingly, c-di-GMP signaling has been shown to be involved in mediating the formation of small colony variants (SCV) in *P. aeruginosa* [82–85]. A phenotypic variant regulator (PvrR), containing a conserved EAL domain, involved in the hydrolysis of c-di-GMP, has been identified to control the wild type-like, antibiotics susceptible revertants [7]. Importantly, the link between antibiotic resistance and biofilm formation was firstly evidenced by the study of such RSCV (rugose small colony variant) phenotypes. The *wspF* mutant, also displays a SCV phenotype and the underlying mechanism was attributed to the activation of a GGDEF domain containing protein WspR [82,83]. Another interesting characteristic of *P. aeruginosa* SCVs with regard to c-di-GMP is the overexpression of Pel and Psl polysaccharides, leading to the auto-aggregation and hyper adherence phenotypes [86–88]. This feature seems to be a common theme for SCV of *P. aeruginosa*.

As we mentioned above, *P. aeruginosa* PAO1 contains 41 DGCs and PDEs that cooperatively mediate the overall concentration of c-di-GMP and finally modulate the EPS production. Original studies have led to extensive functional characterization of c-di-GMP-modulating enzymes and their roles in biofilm formation [7,68,76,77,83,84,88–94]. Based on these results, an emerging model holds that distinct or localized c-di-GMP pools may exist to reciprocally regulate motility and biofilm formation. Different lines of evidences have added bonus points to this hypothesis. One example is *arr* (aminoglycoside response regulator) which is predicted to encode an inner-membrane PDE and seems to be essential for the induction of biofilm formation while contributing to biofilm-specific aminoglycoside resistance [68]. The other example is RoeA, a DGC that plays different roles in regulating motility and biofilm formation as compared to another DGC SadC [94]. Specifically, RoeA contributes to biofilm formation by mainly controlling polysaccharide production, whereas SadC strongly impacts on flagella motility. The studies of these proteins suggested that there were subcellular pools of c-di-GMP in the cell and such pools could be produced from several ways such as specific localization and/or activation of DGCs, limitation of c-di-GMP diffusion through its effectors and/or degraders and the availability of c-di-GMP effectors [71,94].

### 3.2. GacA/GacS Two-Component Systems

**Figure 3:**
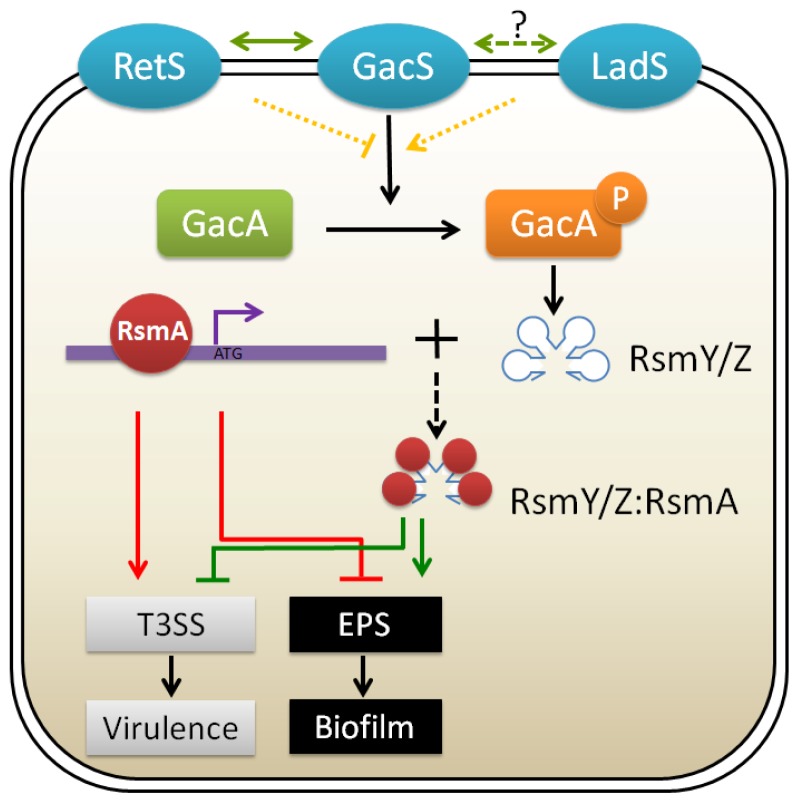
Schematic diagram of the two-component system regulation of biofilm formation and virulence in *P. aeruginosa* [95,96,100,104]. Unknown environmental cues received by the input domains of the three membrane-associated sensor kinases (GacS, LadS and RetS) activate or repress the expression of genes necessary for acute or chronic infection. Free regulatory protein RsmA can bind to the promoter regions of multiple genes, thus repressing expression of biofilm associated genes such as *psl* locus and enhancing bacterial motility and the production of several acute virulence factors (**Red lines**). When the response regulator GacA is phosphorylated by the upstream sensor kinase GacS, the production of small regulatory RNAs RsmZ and RsmY are stimulated, followed by the titrating to RsmA protein, which ultimately de-represses the expression of biofilm-related genes and represses the production of virulence-related factors (**Green lines**). The signaling cascade going through RetS, operating in an opposite manner to that of GacS and LadS, generates more free RsmA, resulting in T3SS activation and biofilm repression (**Red lines**). T3SS, type 3 secretion system. EPS, exopolysacchrides. P means phosphorylated state of GacA. **Dark red circle** indicates RsmA protein, which binds to the genomic DNA without environmental stimulation and binds to RsmY or RsmZ when the upstream pathways are activated.

Expression of the *pel and psl* genes for exopolysaccharide production in *P. aeruginosa* can be regulated by GacA/GacS two-component system. One of the mechanisms involves two histidine kinases, RetS and LadS that act in opposing ways on the GacA/GacS two-component system (Figure 3). The GacA/GacS system subsequently controls the transcription of two small regulatory RNAs (sRNAs), *rsmY* and *rsmZ*, leading to the decrease or increase in the translation of the *pel* or *psl* operon [95,96]. Transcriptomic analysis showed that GacS directly controls the transcription of *rsmY* and *rsmZ*, thereby antagonizing the activities of RNA-binding translational regulator, RsmA, to control the expression of over 500 genes [97,98]. It was further proved that upon binding of RsmA with the promoter of the *psl* operon, the region spanning the ribosome binding site of *psl* mRNA forms a secondary stem-loop structure that prevents ribosome access and the subsequent translation, without affecting transcription [99]. This translational control of Psl expression constitutes a novel example of translational repression by RsmA.

Furthermore, analyses of the mRNA levels using microarray analysis have shown that RetS is required for the expression of genes involved in virulence such as the type III secretion system (T3SS), yet acts as a repressor for the type VI secretion system (T6SS) and genes involved in exopolysaccharide synthesis, leading to the inhibition of biofilm formation [96]. This defines RetS as a pleiotropic regulator of multiple virulence phenotypes that mediates the activation of genes involved in acute infections and the repression of genes associated with chronic persistence [96]. A recent report showed that RetS could directly interact with GacS to modulate its phosphorylation state. [100]. During the acute infection phase, RetS interacts with GacS to form heterodimers, blocking GacS autophosphorylation and leading to reduction in *rsmZ* expression. Finally, RsmA lacking RsmZ will promote the translation of genes required for acute virulence factors. While sensing unknown environmental signals, GacS and RetS each form homodimers, allowing GacS autophosphorylation and subsequent phosphorylation of GacA, finally resulting in the expression of genes involved in chronic infections (Figure 3) [100]. Very recently, a novel RetS interacting protein, PA1611 was identified and characterized as able to both promote biofilm formation and repress T3SS and swarming motility [101], adding complexity to the classical GacS/GacA regulatory cascade. On the other hand, LadS was found to antagonize the effect of RetS, contributing to the repression of T3SS and the activation of genes necessary for exopolysaccharide production promoting biofilm formation [95] (Figure 3). However, there is a paucity of information detailing whether LadS affects the GacS or RetS. Interestingly, one *P. aeruginosa* reference strain PA14 was found to have a natural *ladS* mutation, explaining why PA14 exhibits increased virulence and displays attenuated biofilm formation as compared to PAO1 [102].

In addition, the histidine phosphotransfer (Hpt) protein HptB signaling pathway was found to control biofilm formation and T3SS, and fine-tunes *P. aeruginosa* pathogenesis [103]. Typically, Hpt protein acts as a phosphorylation relay that transits the activation signal from a sensor kinase to the cognate response regulator. Bordi and co-workers found that in contrast to the double control of *rsmYZ* expression by RetS, HptB exclusively regulates *rsmY* expression. Importantly, in this study, they demonstrated a redundant effect of the two sRNAs on T3SS gene expression, while the influence on *pel* gene expression is additive, further underpinning the novel mechanism of fine-tuned regulation of gene expression [103].

### 3.3. Quorum Sensing

Quorum sensing (QS), known as bacterial cell-cell communication system, represents another multicellular activity which involves the production, secretion, and detection of molecules called autoinducers (AIs) to modulate behaviors of the bacterial population [105]. QS provides a strategy for bacteria to detect each other’s presence and to regulate gene expression in response to changes of bacterial population density. Up to now, many biological processes have been found to be controlled by QS, such as bioluminescence, biofilm formation, virulence factor expression, antibiotics production, sporulation, and competence for DNA uptake [106,107].

*P. aeruginosa* employs three quorum sensing signaling systems (LasR/LasI, RhlR/RhlI and PQS) to control cellular processes involved in the production of extracellular virulence factors and to control biofilm formation [108,109]. A large number of genes (as many as 200~300, about 6% of the genome size), including virulence factor genes and genes involved in biofilm development, are activated by two typical, interconnected and homologous acyl-homoserine lactone (AHL) quorum sensing systems, namely the LasR/LasI and RhlR/RhlI systems.

It has been shown that signal molecule 3-oxo-C12-HSL (synthesized by LasI) is necessary for the establishment of a differentiated *P. aeruginosa* biofilm since a *lasI* mutant forms flat, undifferentiated biofilms unlike wild-type biofilms [2] and *lasI* is expressed in a large number of cells during the initial stage of biofilm formation [110]. In contrast, the RhlR/RhlI system was found to be activated during the maturation stage of *P. aeruginosa* biofilm development [111], and might be important for the survival of bacterial cells growing in anaerobic conditions in biofilms [112,113].

In *P. aeruginosa*, quorum sensing regulation of exopolysaccharide was revealed to be mediated by a tyrosine phosphatase named TpbA (PA3885) that is controlled by LasR/I system and negatively regulates Pel polysaccharide production through dephosphorylation of a GGDEF-motif protein, TpbB (PA1120) [114]. This study has generated a common theme that QS seems to be a negative regulator of c-di-GMP signaling. Recently, it was shown that Psl itself could also function as a signaling molecule to stimulate its own expression via two diguanylate cyclases [28], generating a positive feedback circuit that allows efficient biofilm formation. In addition, Gilbert and colleagues found that the QS regulator LasR could bind to the promoter region of the *psl* operon, suggesting that QS can regulate *psl* expression [115]. Furthermore, the release of eDNA was demonstrated to be controlled via AHL- and *Pseudomonas* quinolone signal (PQS)-mediated quorum sensing systems [49].

### 3.4. Other Types of Regulation

**Figure 4:**
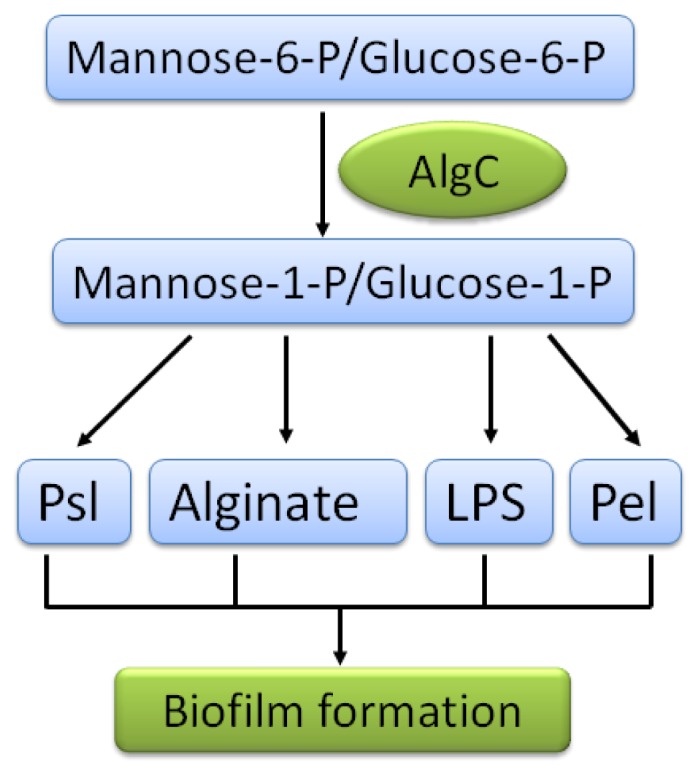
Diagram of AlgC-dependent enzymatic regulation of the production of exopolysaccharides in *P. aeruginosa* PAO1 [116]. The product of *algC* gene (AlgC) is a bifunctional enzyme with phosphomannomutase (PMM) and phosphoglucomutase (PMG) activities that can catalyze the conversion of mannose-6-phosphate (mannose-6-P) and glucose-6-phosphate (glucose-6-P) into mannose-1-phosphate (mannos-1-P) and glucose-1-phosphate (glucose-1-P), respectively. The PMM/PMG activity of AlgC is required for the biosynthesis pathways of four exopolysaccharides (Psl, alginate, LPS and Pel) in *P. aeruginosa*. Experimental data showed that overproduction of individual exopolysaccharides reduces synthesis of the other exopolysaccharides, indicating that AlgC is the checkpoint enzyme that limits the production of *P. aeruginosa* exopolysaccharides, influencing the biofilm formation.

In addition to the typical regulation of biofilm development, the biofilm matrix is also under control by other types of regulation. One example is the metabolic regulation mediated by AlgC, a checkpoint enzyme that coordinates the total amount of exopolysaccharides in *P. aeruginosa* by control of sugar precursors pool for exopolysaccharides synthesis [116] (Figure 4). It was demonstrated that overexpression of one exoplysaccharide could reduce the production of the other. For example, overproduction of Psl led to reduced level of alginate, Pel overexpression resulted in less Psl production, and overproduction of alginate and Psl caused decreased levels of B-band LPS. The enzymatic regulation of exopolysaccharide provided us a very interesting clue about the survival strategy used by *P. aeruginosa* in diverse conditions. It is easily speculated that *P. aeruginosa* produces one major type of exopolysaccharide in certain phases whereas it generates another major type of exopolysaccharide upon changing environments.

As a key polysaccharide for biofilm formation, Psl expression is regulated at multiple levels. In addition to the aforementioned mechanisms, Psl was found to be regulated by RpoS transcriptionally, and post-transcriptionally by RsmA, an RNA binding protein [99]. The transcriptional regulator AmrZ, previously shown to positively regulate twitching motility and alginate synthesis [117], was also shown to directly bind to the promoter region of the *psl* operon to repress its transcription [118]. The AmrZ-mediated switch from Psl production to alginate production provides another example of acute-to-chronic transition strategy used by *P. aeruginosa*.

## 4. Matrix-Driven Strategies against Biofilms

Once biofilms develop into a mature stage, they become extremely difficult to eradicate from infections sites with traditional antimicrobial agents [6]. Agents that inhibit biofilm formation or transform bacteria from biofilm life style to free-living individuals are ideal to eradicate biofilm. The strategies used for anti-biofilm mainly stem from two basic ways: matrix synthesis and its regulatory mechanisms.

For example, disruption of the initial attachment that is dependent on a large array of adhesins would contribute to inhibition of the establishment of biofilms, while the digestion of the EPS matrix may be another method to interfere with biofilm formation. As we mentioned before, DNase I treatment has already shown efficacy in the inhibition of the early development of biofilm [50]. It was also reported that alginate lyase could enhance antibiotic killing of mucoid *P. aeruginosa* in biofilms [119]. In addition, the macrolide antibiotic azithromycin was shown to block alginate formation and quorum sensing signaling [120] and was further reported to improve lung function of CF patients, especially in the subgroup chronically colonized by *Pseudomonas* [121].

Antagonizing the intracellular signaling molecules to control biofilm formation has also been investigated. One example is the identification of furanones, which have shown their ability to inhibit the biofilm formation of *P. aeruginosa in vitro* [122,123]. Molecules of this type have been reported to function through inhibiting the AHL-dependent QS systems in *P. aeruginosa*. Iron has also been employed in distinct aspects to control the formation of biofilms. Singh and his colleagues have identified an innate immunity component, lactoferrin, which prevents *P. aeruginosa* biofilm formation by chelating iron and stimulating the type IV pili-mediated twitching motility [124]. Furthermore, iron salts such as ferric ammonium citrate were found to not only perturb biofilm formation but also disrupt existing biofilms by *P. aeruginosa* [125]. In a screen of co-therapy of antibiotics against *P. aeruginosa*, 14-alpha-lipoyl andrographolide (AL-1), a diterpenoid lactone derivative from the herb *Andrographis paniculata* appeared to inhibit biofilm formation by decreasing EPS production and to sensitize the bacterium to a variety of antibiotics [126].

Recently, it was found that Gram-positive bacterium *Bacillus subtilis* produced a factor that prevented biofilm formation and could break down existing biofilms. The factor was identified to be a mixture of d-leucine, d-methionine, d-tyrosine, and d-tryptophan that could disassemble at nanomolar concentrations. d-amino acid treatment subserved the release of amyloid fibers that linked cells together in the biofilm. In addition, d-amino acids also prevented biofilm formation by *Staphylococcus aureus* and *P. aeruginosa*, indicating it may be a widespread signal for biofilm disassembly [127]. Furthermore, the same group identified another biofilm disassembly compound, norspermidine, which targets directly and specifically with the exopolysaccharide matrix and this biofilm inhibition effect could be enhanced together with d-amino acids and is effective in other bacterial species [128].

## 5. Perspectives

Accumulating data presented in the recent literature provides valuable insights into the novel roles of the biofilm matrix and its regulatory mechanism in *P. aeruginosa* biofilm formation. A deep understanding of the mechanisms involved in biofilm formation will ultimately shed light on the generation of alternative treatments for *P. aeruginosa* infections. There is no doubt that future studies will reveal additional biofilm matrix components and identify more elaborate regulatory circuits for biofilm formation. Finally, the interaction of the biofilm matrix and the synergistic effects of different anti-biofilm strategies should also be regarded as major concerns.

## Figures and Tables

**Table 1:** Roles of different exopolysaccharides (EPS) in *Pseudomonas aeruginosa*

EPS	Locus *	Roles	References
Psl	PA2231–PA2245	Initial attachment and adhesion	[15,17–19]
		Primary biofilm scaffold	[15,19]
		Proinflammatory signaling	[20]
		Antibiotics resistance	[19]
		Avoidance of host defence mechanisms	[30]
		Signaling molecule to stimulate biofilm formation	[28]
		Resistance to biofilm inhibitor Polysorbate 80	[31]
		Guide of exploration and microcolony formation	[26]

Pel	PA3058–PA3064	Pellicle formation and solid surface-associated biofilm formation	[11]
		Aggregating of bacterial cells	[14]
		Aminoglycosides antibiotic resistance	[14]
		Initial attachment in the absence of type IV pili	[34]

Alginate	PA3540–PA3548	Persistence and immune evasion	[44]
		Resistance to antibiotics as well as opsonophagocytosis	[45,46]
		ROS scavenge	[47]
		Leading to mucoid	[39]
		Water and nutrient retention	[41]

eDNA		Nutrient	[54,55]
		Scaffold	[49]
		Antibiotics resistance	[53]
		Major proinflammatory component	[57]
		Promoting self-organization of bacterial biofilms	[58]

Proteinaceous component	Flagella	Initial cell-to-surface interactions	[60]
	Pili	The formation of mushroom-like microcolony	[60]
	CdrA	Mediate aggregation and increase biofilm stability	[63]
	Cup fimbriae	Cell-to-cell interaction and microcolony formation	[66,67]

* All loci were extracted from *Pseudomonas* Genome Database (www.pseudomonas.com) [21]. ROS, reactive oxygen species.
